# Effectiveness of online caries management platform in children's caries prevention: A randomized controlled trial

**DOI:** 10.3389/fpubh.2023.1102503

**Published:** 2023-02-09

**Authors:** Siqi Yan, Song Luo, Xiaoxia Yang, Lidan He, Xinyi Chen, Guoying Que

**Affiliations:** Stomatological Hospital, Southern Medical University, Guangzhou, China

**Keywords:** the internet, caries management, caries risk, effectiveness, school children

## Abstract

**Purpose:**

To construct an online caries management platform and evaluate its efficacy in children's caries prevention based on caries risk.

**Methods:**

The study participants were second-grade pupils. The caries risk assessment tool (CAT) was used to grade caries risk for all participants, who were randomly divided into the experimental (114 pupils) and control (111 pupils) groups. The experimental group used the Internet for caries management, while the control group was managed by traditional lecturing in classroom. The caries status of each surface of the first permanent molars was recorded. The basic information and oral health knowledge, attitude, and behaviors of participants were collected by questionnaire. One year later, outcome data were collected. Pearson's chi-squared test was used to analyze the caries risk assessment items and oral health behaviors. The Mann-Whitney *U*-test was used to analyze the decayed-missing-filled surfaces (DMFS) index, plaque index, and scores of oral health knowledge and attitude. *P* < 0.05 was considered statistically significant. This study was available on the website of Chinese Clinical Trials Register (No: MR-44-22-012947).

**Results:**

After 1 year, the oral health knowledge score was improved by 20.58% (*P* < 0.001) in the experimental group and 6.02% in the control group. The plaque index was improved by 49.60% (*P* < 0.001) in the experimental group and 21.01% in the control group. The DMFS index increased in both groups but there were no significant differences (*P* = 0.608). The experimental group had a better improvement effect in caries risk assessment items than the control group, including “whether the frequency of eating sugary snacks or drinks between meals is more than 3 times/day” (*P* = 0.033) and the use of fluoridated toothpaste (*P* = 0.020). The experimental group was better than the control group in reported oral health behaviors, including frequency of eating sweets before sleep (*P* = 0.032), brushing time (*P* = 0.001), and the filled rate (proportion of FS in DMFS) of first permanent molars (*P* = 0.003).

**Conclusions:**

The online caries management platform showed more advantages than traditional lecturing in improving oral health knowledge and behaviors (oral hygiene practice, sugar consumption behavior, and medical treatment behavior). This platform provides a reliable implementation path for the occurrence and continuous improvement of oral health-related behaviors.

## 1. Introduction

In the past 10 years, with the rapid economic development of Guangdong Province, China, the government has invested a large amount of manpower and material resources in the prevention and treatment of children's caries. However, the prevalence of children's caries is still increasing (1). The caries-free rate of primary teeth of children aged 5 years (21.6%) was far lower than the World Health Organization's (WHO) target of a 50% caries-free rate, and the filled rate of dental caries was only 1.3% (2). Therefore, as effect of caries prevention is not ideal, it is necessary to develop a new model for children's oral health.

Caries is a chronic disease that can be prevented, treated, and managed. Chronic disease self-management includes both interventions provided by healthcare providers and healthy behaviors that patients adopt. This requires patients to have the skills to make informed and autonomous decisions, make self-goals, monitor themselves, acquire and utilize disease-related knowledge, communicate with doctors, and manage their emotions (3). In many developed countries, patients have been gradually transferred from passive treatment to self-management of chronic diseases (4, 5), and the effectiveness and importance of self-management have been recognized. Web-based chronic disease self-management is a platform to provide patients with disease-related self-behavior management through the Internet, including the development of self-management goals, personal health promotion behavior records, and disease-related knowledge and skills education. It is effective to guide the self-management behavior of patients with chronic diseases through the Internet, remote video and other technologies (6–9). The literature review of Omboni et al. (10) showed that after remote health intervention, most disease-related physiological indicators were improved, risk factors were controlled, lifestyle was positively changed, and the quality of life and patient compliance were improved. In the field of oral health care, An app called “Brush DJ” effectively motivated the oral health management of 189 participants (11). For another example, the mobile application “White Teeth” was developed for patients wearing orthodontic devices to help improve their oral care behavior and oral hygiene (12).

Chronic disease management of caries is a coordinated healthcare intervention system in which patient self-care efforts are important. Chronic disease management of childhood caries aims to improve the responsibility of children and their guardians for their health. The traditional model of dental care delivery needs to evolve into a collaborative partnership between oral health service providers and patients and their families. Because dental caries is a chronic disease, which is significantly affected by social and behavioral factors, effective management requires individual self-management of cariogenic factors. An important role of the professional team is to provide guidance and support to patients and their families to make necessary lifestyle changes, such as oral hygiene practices, dietary habits, and fluoride use. This personalized patient care approach is the essence of the chronic disease management model (13). Chronic disease management of children's caries is a hierarchical, family-centered model (14), which can reduce modifiable cariogenic factors and reduce the rate of new-onset caries. The content of hierarchical management of children's caries is mainly to carry out personalized caries prevention and treatment after caries risk assessment (13). Abanto et al. (15) observed a caries prevention program for children based on caries risk assessment for 2 years, and the results showed that this program could effectively prevent early caries and control active caries in children. Yeo and Lee (16) conducted caries management using mobile application for preschoolers, and it had effects on dental plaque, mineral loss and cariogenic bacteria, especially for children in the extreme high- risk group and the high- risk group.

A recently introduced online caries management platform can evaluate the caries risk of children and systematically manage their dental caries. This wechat public account is called “Guangdong Caries Prevention and Control,” which is based on a caries risk assessment tool published by the American Academy of Pediatric Dentistry (AAPD) (17). It has a patient-centered, risk-based, evidence-based caries management, and achieves doctor-patient interactive caries management. This study intended to conduct caries management for 1 year among second grade pupils who were 6–8 years old at baseline. By evaluating the effect of caries management based on the online caries management platform compared with traditional lecturing management in the classroom, we aimed to provide theoretical guidance for the management and prevention of children's caries.

## 2. Methods

### 2.1. Trial design and study participants

**Figure 1:**
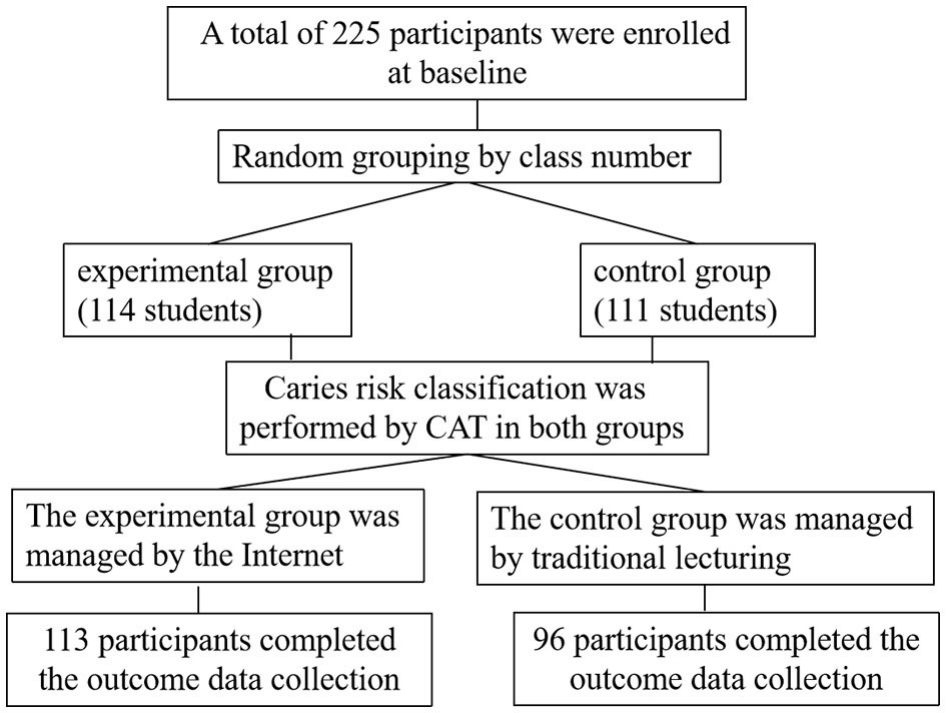
Flowchart of the study.

The trial was a randomized controlled trial with two parallel groups (experimental and control) and data measurements at two time points (baseline and after 1 year). Cluster sampling method was adopted in this study. Among the five schools that could be contacted, one school was selected as the sample population of this study. The number of second-grade students in this school had met the required sample size. Complete data were obtained from 225 students at baseline. Students from 6 classes were randomly divided into the experimental group and the control group. There were 114 students (62 boys and 52 girls) in the experimental group and 111 students (59 boys and 52 girls) in the control group. One year later, complete outcome data were collected for 113 students (61 boys and 52 girls) in the experimental group and 96 (50 boys and 46 girls) in the control group. The loss to follow-up rate was 0.88% in the experimental group and 13.51% in the control group, respectively. During 1 year of follow-up, 13 participants in the control group asked to leave the study. When the outcome data was collected, one participant in the experimental group and two participants in the control group were absent. The effective sample size was 209 students, as shown in Figure 1.

### 2.2. Ethical considerations

The study passed ethical review by the Ethics Committee of Stomatological Hospital, Southern Medical University (Approval No: [2021] 43). The study was available on the website of the Chinese Clinical Trials Register (medical research record No: MR-44-22-012947). This study was conducted in accordance with the Declaration of Helsinki. Official permission was obtained from the concerned school authorities before starting the study. Written informed consent was obtained from all parents of the study participants and verbal assent from each child.

### 2.3. Eligibility criteria

Inclusion criteria: second-grade students aged 6–8 years at baseline, able to cooperate with the oral examination and questionnaire survey, no history of mental illness, no history of systemic diseases, no history of long-term drug use, no history of allergy, no participation in other programs, and able to continue to participate in this program within 1 year. The legal guardians of the children in the study had WeChat accounts and electronic devices with Internet access and were able to cooperate with the researchers to complete relevant online research projects. The legal guardians of the children in the study had a good understanding of the study and the willingness to sign relevant informed consent forms.

Exclusion criteria: children with intellectual or mental illness, children with severe physical disabilities, children who were unable to cooperate with the examination, and children who were ready to transfer to another school within 1 year.

### 2.4. Randomization

In consideration of the practical feasibility of the experiment, the intervention method must be kept consistent in each class to avoid pollution, so we carried out random grouping by class number. A randomization master list was compiled from computer-generated random numbers, and each class was assigned to a group (experimental group, control group) by the biostatistician, using opaque envelopes to hide the assignment results.

To ensure comparability of baseline information between the two groups, the baseline data of the two groups were statistically analyzed after grouping, and it was found that there was no significant difference between the two groups.

### 2.5. Blinding

Because of the nature of the interventions, neither children nor research practitioners were masked to the intervention. To reduce the bias, the personnel involved in data collection and statistical analysis were blinded in this study.

### 2.6. Sample size

This study considered (95% CI) and 80% power to estimate the sample size. Mean ± SD (plaque index) value of intervention group (1A) 0.87 ± 0.35 and mean ± SD value of control group (1B) 1.07 ± 0.37 respectively were taken (18). Therefore, mean of control group (μ1) = 1.07, mean of intervention group (μ2) = 0.87 and average standard deviation of control and intervention group (ɓ) = 0.36. Using the following formula (19): Sample Size (n) = [2 ɓ^2^ (z_α/2_+z_β/2_)^2^]/(μ1–μ2)^2^, the sample size was calculated as 51. Estimating a 20% loss to follow-up rate, we calculated the theoretical sample size of each group as 64 participants, with a total of 128 participants required.

### 2.7. Questionnaire

Parents of children in the experimental group searched the WeChat Service named “Guangdong Caries Prevention and Control” on their mobile phones to register and fill in the basic information. The researchers provided real-time instructions to fill in the electronic questionnaire online. The questionnaire system was set up to automatically identify whether the questionnaire items were filled out completely and automatically find out the possible logical errors, which could be checked again by the researchers.

Trained researchers conducted face-to-face questionnaire interviews with parents of children in the control group.

The questionnaire was derived from the Fourth National Oral Health Survey (20), which was designed based on previous studies to identify factors related to dental caries in children and was proved to be with appropriate reliability and validity before the study. An 8-item interview was conducted to assess oral health knowledge and a 4-item interview was used to assess oral health attitude. For each question, you got 1 point for a correct answer and 0 points for a wrong answer. A composite score was then generated by adding the individual scores together. During the questionnaire survey in both groups, we patiently explained the work to reassure parents and deal with potential bias.

The basic information of the study participants was kept strictly confidential and subjected to the supervision of the ethics committee.

### 2.8. Clinical examination

The caries examination standard of the WHO (21) was used to record the caries lesions. The physicians who performed the oral examination included one senior physician (a dentist with more than 10 years of clinical experience and a license for medical practitioner) and three resident doctors (a dentist with more than 2 years of clinical experience and a license for medical practitioners). Three resident doctors received standardized caries examination methods and skills training by using clinical intraoral photographs. Each resident doctor independently examined 20 children aged 6–8 years who were not included in the study. The senior physician rechecked the examination results of each resident doctor and conducted reliability tests for each resident doctor and among resident doctors. The procedure was repeated until the intra-examiner kappa values and inter-examiner kappa values of the three resident doctors were >0.85. A Community Periodontal Index (CPI) probe (Kangqiao Company, China) and a plane mirror (Kangqiao Company, China) with an Light Emitting Diode (LED) light (Vogel Shanghai Technology Co., LTD, China) were used. Dental caries was diagnosed at the cavitation level and verified by the ball-end CPI probe.

In the formal examination, after all the teeth were examined according to WHO standards and the checklists were completed, 5% of the participants were randomly selected for repeated examination to compare the reliability between the examiners. The inter-examiner kappa values were 0.89 (examiner 1 to examiner 2), 0.88 (examiner 2 to examiner 3), and 0.86(examiner 3 to examiner 1).

Plaque index (PLI) determination: The modified Silness-Loe plaque index (22) was used to examine the labial surfaces of four maxillary incisors (the plaque of permanent teeth with eruption was recorded; if permanent teeth did not erupt, the plaque of primary teeth was recorded). The average plaque index of four incisors labial surfaces of the participant was used as the plaque index score of the participant (23).

Paraffin-stimulated whole saliva was collected over 5 min and salivary secretion rate was determined.

The caries risk in both groups was assessed according to the CAT proposed by the AAPD.

### 2.9. Intervention

**Figure 2:**
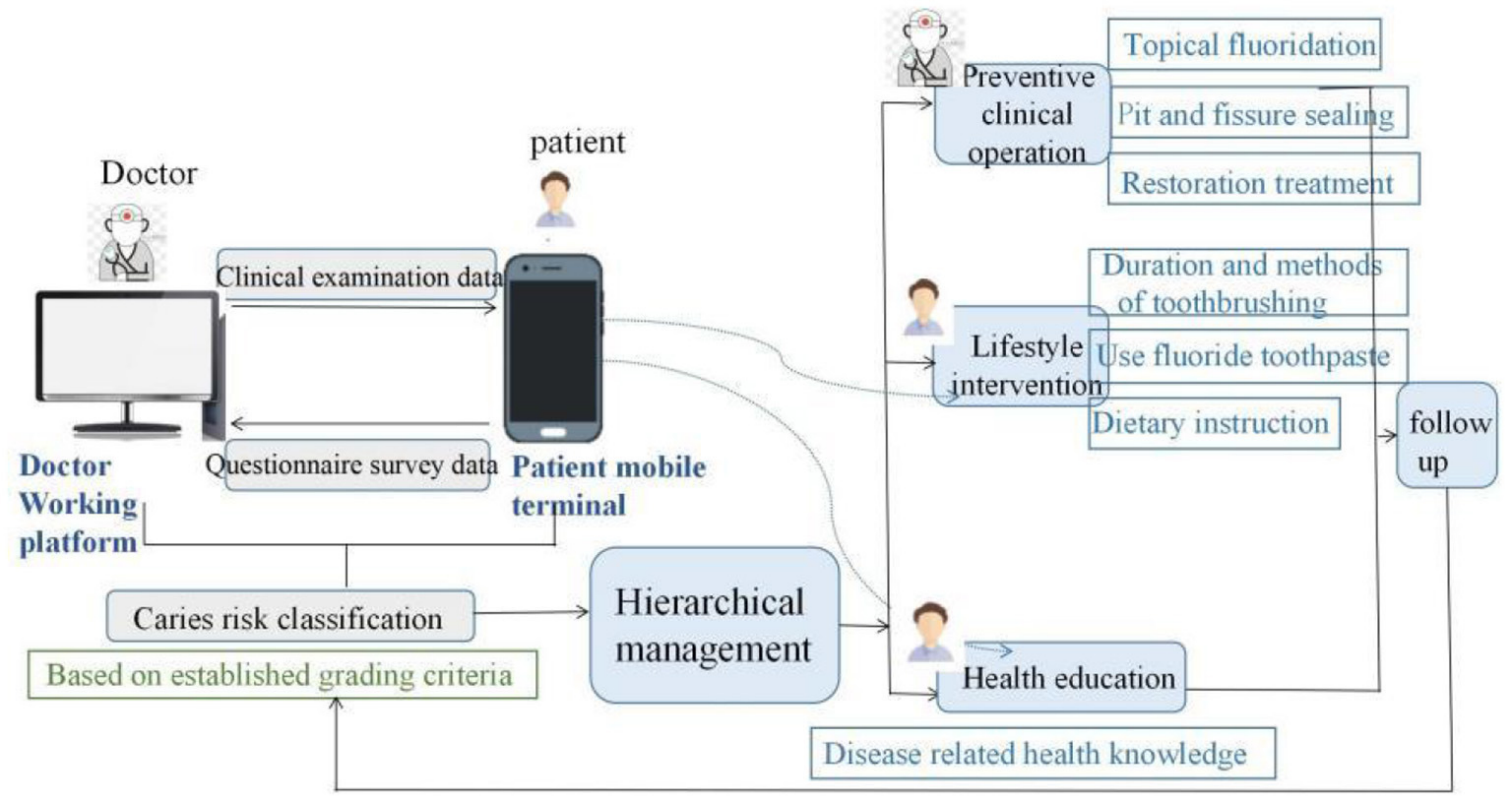
Schematic diagram of the online intervention process of the experimental group.

**Figure 3:**
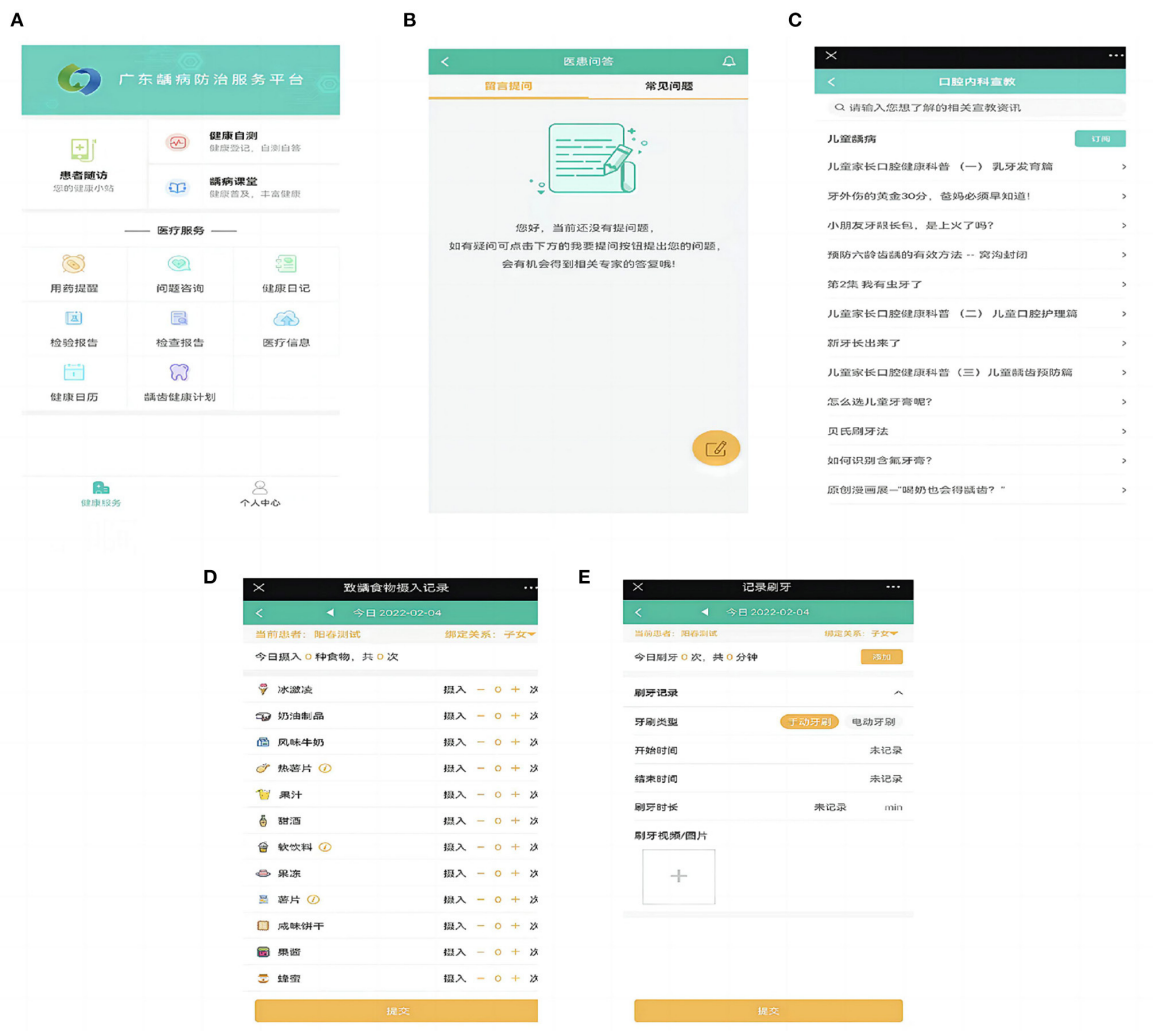
Functions of patients' mobile terminal. **(A)** Main interface of patients' mobile terminal; **(B)** Doctor-patient question answering system; **(C)** Health education; **(D)** Diet monitoring; **(E)** Teeth brushing recording.

Caries management measures of different intensities would be implemented according to caries risk level (17). Through the WeChat public platform of “Guangdong Caries Prevention and Control,” the doctor side sent the clinical examination data of the study participants to the patient side, and the patient side fed back the questionnaire data to the doctor side. After data integration, caries risk was graded for the study participants, and then hierarchical management was conducted. The experimental group performed pit and fissure sealing (3M company, the United States) at school. Caries restoration treatment was carried out at their own expense according to the actual situation of the children and their parents. Topical fluoridation (Colgate, the United States, 5% sodium fluoride) at school every 3 months for high-risk children, every 6 months for intermediate-risk children, and every 12 months for low-risk children. Participants in the experimental group were required to regularly access the WeChat public platform to receive self-management internet intervention at least every 3 months for high-risk children, at least every 6 months for intermediate-risk children, and at least every 12 months for low-risk children. The self-management internet intervention mainly included the following contents: Researchers assisted the study participants to develop specific and personalized self-management goals. The study participants completed caries online health education, diet guidance, online brushing, fluoride use (fluoridated toothpaste ingredient list) uploading, and online doctor-patient interaction. Participants who did not actively use the platform were given reminders. The purpose of caries management was achieved by using the online caries management platform, as shown in Figure 2. Figure 3 shows functions of the patients' mobile terminal.

After collecting clinical examination data and questionnaire data of control group. Caries risk was also graded for participants in the control group, and then hierarchical management was conducted. In addition to offline preventive clinical operations (topical fluoridation, pit and fissure sealing) with the same frequency as the experimental group, the researchers also conducted regular interventions for children in the control group in classroom, including oral health education, diet instruction, teaching children how to choose and use fluoridated toothpaste, education on brushing methods, and encouraging children with caries to seek medical treatment in time. The interval of interventions was every 3 months for those at high risk, every 6 months for those at intermediate risk, and every 12 months for those at low risk.

### 2.10. Outcomes

The primary outcome measures were changes in DMFS index, plaque index, caries risk assessment items, and oral health-related knowledge, attitude, and behavior (oral hygiene practice and sugar consumption behavior) after intervention at 1 year in the experimental and control groups.

### 2.11. Statistical analysis

After completion of the trial, the obtained data were entered in Epi Data 3.0 and analyzed using Statistical Product and Service Solutions (SPSS) (IBM company, the United States, version 23.0). The level of significance was set at *P* < 0.05.

Based on the results of the normality test and homogeneity of variance test, parametric analysis or non-parametric analysis methods were selected:

Pearson chi-square test was used to analyze parents' diploma degree, family income, whether one-child, caries risk assessment items, oral health-related behavior, and caries filled rate in two groups before and after a year. Fisher exact probability method was used when the sample size was <40 or the theoretical frequency was <1. For row × list data, if the theoretical frequency of more than 1/5 grids was <5, or one theoretical frequency was <1, the likelihood-ratio test could be used.

The Mann-Whitney U test was used to analyze the DMFS index of first permanent molars, plaque index, and the score of oral health knowledge and attitude in two groups before and after 1 year.

## 3. Results

### 3.1. Basic information

The caries management methods of the two groups were different. Being an only child, parents' diploma degree, and the average monthly family income were detected as potential influencing factors for the effect of different caries management methods. Therefore, the above variables at baseline in the two groups were statistically analyzed. Differences were found to not be statistically significant. *P*-values of the above variables were 0.607, 0.976, 0.570, and 0.439 respectively, as shown in Table 1.

**Table 1:** Baseline information of the two groups (%).

**Factors**	**Group**	**Experimental group**	**Control group**	**χ^2^**	***P***
Whether the child is an only child	Yes	8.77	10.81	0.265	0.607
	No	91.23	89.19		
The father's education level	Lowest	2.63	1.80	0.210^a^	0.976
	Low	25.44	26.13		
	Moderate	35.09	36.04		
	High	36.84	36.04		
The mother's education level	Lowest	2.63	2.70	2.010^a^	0.570
	Low	28.95	32.43		
	Moderate	29.82	35.14		
	High	38.60	29.73		
Average monthly household income	<5,000 RMB	35.96	42.34	1.647	0.439
	5,000–10,000 RMB	50.88	42.34		
	>10,000 RMB	13.16	15.32		

a Likelihood-ratio test.

### 3.2. Caries risk assessment items

Table 2 shows that there were no significant differences in high, medium, and low caries risk assessment items between the two groups at baseline (*P*-values of these variables were >0.05). After 1 year, the incidence of “eating sugar-sweetened snacks or drinks between meals >3 times/day” decreased in both groups, and the difference was statistically significant (*P* = 0.033, χ^2^: 4.569). “Brushing their teeth with fluoridated toothpaste every day,” the incidence of this item increased in both groups, and the difference between the two groups was statistically significant (*P* = 0.020, χ^2^: 5.934). The fluoride content of toothpaste in the experimental group was determined by uploading the toothpaste ingredient list on the online caries management platform by children's parents. In the control group, the fluoride content of toothpaste was determined by taking the toothpaste to the investigation site. The toothpaste ingredient list uploading function of the platform provided great convenience for researchers to guide participants to buy fluoridated toothpaste. The online caries management showed a better effect on the improvement of the changeable caries risk assessment items than the control group.

**Table 2:** Caries risk assessment items in two groups before and after a year (%).

**Caries risk items**	**Baseline**				**After 1 year**			
	**Experimental group**	**Control group**	**χ^2^**	***P***	**Experimental group**	**Control group**	**χ^2^**	***P***
***Risk factors, social/biological***								
Patient has a lifetime of poverty, low health literacy	25.44	30.63	0.752	0.386	24.78	31.25	1.804	0.298
Patient has frequent exposure (>3 times/day) between meal sugar-containing snacks or beverages per day	27.19	31.53	0.511	0.475	15.93	28.13	4.569	0.033^*^
Child is a recent immigrant	0.88	2.70	1.327^a^	0.249	0	0		
Patient has special health care needs	0.88	1.80	0.478^a^	0.489	0.88	2.08	0.527^a^	0.595
***Protective factors***								
Patient receives optimally fluoridated drinking water	0	0			0	0		
Patient brushes their teeth daily with fluoridated toothpaste	44.74	52.25	1.272	0.259	78.76	63.54	5.934	0.020*
Patient receives topical fluoride from a health professional	20.18	20.72	0.010	0.919	92.98	93.69	0.046	0.831
Patient has dental home/regular dental care	5.26	5.41	0.002	0.962	10.62	8.33	0.313	0.576
***Clinical findings***								
Patient has ≥1 interproximal caries lesions	86.84	87.39	0.015	0.903	87.61	86.49	0.061	0.805
Patient has active non-cavitated (white spot) caries lesions or enamel defects	13.16	18.92	1.389	0.239	13.27	11.46	0.157	0.692
Patient has a low salivary flow	0.88	0	1.364^a^	0.243	2.65	2.08	0.073^a^	1.000
Patient has defective restorations	6.14	11.71	2.156	0.142	2.65	6.25	1.628^a^	0.307
Patient wears an intraoral appliance	0	0			0	1.04	1.183^a^	0.459

* Statistically significant at P < 0.05,

a Fisher's exact probability method.

### 3.3. Caries related behaviors

There were no significant differences in the four sugar consumption behaviors involved in this study between the two groups at baseline. An additional table shows this in more detail (see Supplementary Table 1). Table 3 shows that there were no significant differences in the frequency of eating dessert (*P* = 0.852, χ^2^:1.981), drinking a sweet drink (*P* = 0.450, χ^2^: 4.730), and drinking sweet milk (*P* = 0.540, χ^2^: 4.067) at ordinary times between the two groups after a year, but there were significant differences in the frequency of eating dessert or drinking a sweet drink before bedtime (*P* = 0.032, χ^2^: 6.885), the experimental group had a low frequency of sugar intake before bed.

**Table 3:** Sugar consumption behavior of the two groups after a year (%).

**Factors**	**Group**	**After a year**		**χ^2^**	***P***
		**Experimental group**	**Control group**		
Does your child eat dessert or drink sweet drinks before bed?	Every day	12.39	23.96	6.885	0.032^*^
	Not every day	70.80	67.71		
	Seldom	16.81	8.33	
Frequency of eating desserts and sweets	≥2 times a day	1.77	4.17	1.981^a^	0.852
	Once a day	5.31	7.29		
	Once a week	16.81	16.67		
	2–6 times a week	29.20	30.21		
	1–3 times a month	11.50	8.33		
	Rarely	35.40	33.33	
Frequency of drinking sweet drinks (carbonated drinks, not freshly squeezed fruit juices)	≥2 times a day	1.77	1.04	4.730^a^	0.450
	Once a day	1.77	1.04		
	Once a week	13.27	16.67		
	2–6 times a week	6.19	10.42		
	1–3 times a month	13.27	19.79		
	Rarely	63.72	51.04	
Frequency of drinking sweetened milk, yogurt, soy milk, milk powder, tea, coffee	≥2 times a day	1.77	1.04	4.067^a^	0.540
	Once a day	11.50	18.75		
	Once a week	7.96	10.42		
	2–6 times a week	16.81	11.46		
	1–3 times a month	10.62	7.29		
	rarely	51.33	51.04	

* Statistically significant at P < 0.05,

a Likelihood-ratio test.

At baseline, there were no significant differences in the four oral hygiene behaviors involved in this study between the two groups. An additional table shows this in more detail (see Supplementary Table 2). After a year, Table 4 shows that there was a significant difference between the two groups in “brushing time” (*P* = 0.001, χ^2^: 14.038). The online caries management platform provided the brushing time countdown function, which was of great help to improve the brushing time of the experimental group. The oral health behaviors of the two groups developed in a good direction. The online caries management platform had greater advantages in improving oral hygiene behaviors than traditional lecturing.

**Table 4:** Oral hygiene behavior of the two groups after a year (%).

**Factors**	**Group**	**After a year**		**χ^2^**	***P***
		**Experimental group**	**Control group**		
Frequency of using dental floss	Every day	4.42	2.08	1.334^a^	0.721
	Every week	11.50	10.42		
	Occasionally use	41.59	39.58	
	Don't know or never use it	42.48	47.92		
Brushing times per day	≥2 times per day	62.83	63.54	1.198^a^	0.549
	Once a day	35.40	32.39		
	<1 time a day	1.77	4.17	
Time of brushing each time	>3 min	31.86	10.42	14.038	0.001^*^
	1–3 min	42.48	58.33		
	<1 min	25.66	31.25	

* Statistically significant at P < 0.05,

a Likelihood-ratio test.

At baseline, there was no significant difference in plaque index between the two groups (Z: −1.313, *P* = 0.189). The plaque index decreased by 49.60% (Z: −6.582, *P* < 0.001) in the experimental group in comparison to 21.01% in the control group. Online caries management platform had advantages in improving oral hygiene quality, as shown in Table 5.

**Table 5:** Plaque index in two groups at baseline and after a year (mean ± SD).

**Plaque index**	**Experimental group**	**Control group**	**Z**	***P***
Baseline	1.25 ± 0.64	1.38 ± 0.67	−1.313	0.189
After 1 year	0.63 ± 0.45	1.09 ± 0.53	−6.582	<0.001^*^
% Change	49.60	21.01		

* Statistically significant at P < 0.05, SD, Standard Deviation.

At baseline, there was no significant difference in the caries filled rate of the first permanent molars between the two groups (*P* = 0.310, χ^2^: 1.029). One year later, the caries filled rate of the first permanent molars in the experimental group and the control group increased to 69.32 and 46.99%, respectively, and the difference between the two groups was statistically significant (*P* = 0.003, χ^2^: 8.772), as shown in Table 6.

**Table 6:** The caries filled rate of first permanent molars in two groups before and after a year (%).

**Caries filled rate**	**Experimental group**	**Control group**	**χ^2^**	***P***
Baseline	19.15	11.76	1.029	0.310
After 1 year	69.32	46.99	8.772	0.003^*^

* Statistically significant at P < 0.05.

### 3.4. Oral health knowledge and attitude

As shown in Table 7, there were no significant differences in the scores of oral health knowledge (Z: −1.780, *P* = 0.075) and attitude (Z = −0.081, *P* = 0.935) between the two groups at baseline. After 1 year, the oral health knowledge score was improved by 20.58% (Z: −4.418, *P* < 0.001) in the experimental group in comparison to 6.02% in the control group. The scores of oral health attitude of the experimental group and the control group were improved, but the difference was not statistically significant (Z: −0.062, *P* = 0.951).

**Table 7:** Oral health knowledge and attitude in two groups at baseline and after a year (mean ± SD).

**Knowledge and attitude**	**Experimental group**	**Control group**	**Z**	***P***
**Oral health knowledge**				
Baseline	5.88 ± 1.28	6.15 ± 1.26	−1.780	0.075
After 1 year	7.09 ± 0.84	6.52 ± 0.94	−4.418	<0.001^*^
% Change	20.58	6.02		
**Oral health attitude**				
Baseline	3.89 ± 0.34	3.88 ± 0.49	−0.081	0.935
After 1 year	3.95 ± 0.22	3.92 ± 0.31	−0.062	0.951
% Change	1.54	1.03		

* Statistically significant at P < 0.05, SD, Standard Deviation.

### 3.5. Caries status

Table 8 shows that there were no significant differences in DMFS of first permanent molars at baseline (Z = −0.983, *P* = 0.326) and after a year (Z = −0.514, *P* = 0.608). At baseline, the caries prevalence of the first permanent molar was 24.32% in the control group and 18.42% in the experimental group, with no significant difference (*P* = 0.280, χ^2^: 1.168). After 1 year, the caries prevalence of the first permanent molar was 33.33% in the control group and 30.97% in the experimental group, with no significant difference (*P* = 0.761, χ^2^: 0.133).

**Table 8:** DMFS of first permanent molars at baseline and after a year (mean ± SD).

**DMFS**	**Experimental group**	**Control group**	**Z**	***P***
Baseline	0.42 ± 1.17	0.53 ± 1.17	−0.983	0.326
After a year	0.78 ± 1.78	0.87 ± 1.67	−0.514	0.608
% Change	85.71	64.15		

DMFS, decayed-missing-filled surfaces index; SD, Standard Deviation.

## 4. Discussion

The results of this study suggest that the online caries management platform has more advantages in caries risk items, oral health knowledge and oral health behaviors than the traditional lecturing.

The online caries management platform provides a reliable implementation path for the occurrence and persistence of health-related behaviors. The significant increase in the use rate of fluoridated toothpaste and the extension of brushing time in the experimental group were mainly attributed to the functional modules of toothpaste ingredient list uploading, brushing time recording and personalized health education on the online caries management platform. A six-year oral health promotion programme carried out in school for children in Wuhan city, PR China suggested that use rate of fluoridated toothpaste was improved to 27.47% (24), while the use rate of fluoridated toothpaste in experimental group of this study increased from 44.74 to 78.76%. The online caries management platform can improve the accuracy and convenience of toothpaste ingredient judgment by uploading ingredient list. An app also effectively motivated 88% participants to brush their teeth for longer (11). However, the platform of this study has a defect that cannot monitor the authenticity of participants' uploaded brushing time, which will be improved in future studies. The decrease in sugar consumption frequency in the experimental group was due to the procedure of cariogenic food intake recording provided by the platform and the online caries education. A previous study showed that high frequency bedtime sweet consumption mainly contributed to caries of the 5-year-old children in Shandong, China (25). The online caries management platform can effectively reduce the frequency of bedtime sweet consumption. The significant increase in caries filled rate in the experimental group indicated that the online caries management platform could improve the participants' attention to caries treatment. Previous studies have shown that oral health education can improve the oral hygiene quality of participants (26–28), and the plaque index is often used as an objective and quantitative evaluation index. In this study, the plaque index of the control group decreased after 1 year, while the plaque index of the experimental group decreased more significantly. This may be because the experimental group used the functional module of brushing time recording through the online caries management platform, and the participants with poor oral hygiene at baseline received online education on intensive brushing methods. The improvement of above oral health behaviors will need to be observed over a longer period of time in future studies.

Oral health knowledge and attitude improved after 1 year in both groups, but not by much from the baseline. It is relatively easy to improve health knowledge and attitude, but it is difficult to change health behavior (29), and the online caries management platform has more advantages. It is a complex and difficult process for people to put into action what they know and believe. Only by transforming the firmly believed health knowledge into behavior can health promotion results be achieved. It is an important task of health education to promote the consistency of health knowledge and health behavior, which is also the difficulty of health education. The online caries management platform is more advantageous to achieve this important task. The Information Motivation Behavioral Skills Model (IMB) was originally developed for the intervention of high-risk behaviors of AIDS. Information, motivation, and behavioral skills are three factors that influence and interact with each other in health behavior change. The application of the IMB model in the behavioral intervention of diabetes and obesity patients achieved good results (30, 31). Vernon et al. (32) used the IMB model to conduct preventive oral health education for HIV-positive patients and found that the oral health behaviors of the participants changed significantly. Obtaining information through health education is a necessary but not sufficient condition for generating healthy behaviors. The traditional health education model simply transmits health-related information to the target population, which has a certain effect on promoting the occurrence of health behaviors, but the effect is limited, and the effect on the health-related motivation and behavioral skills of the target population is weak.

Health-related information and motivation can activate behavioral skills and motivate the target population to initiate and sustain healthy behaviors. Early adolescence is a life stage in which many health behaviors are perpetuated. Health interventions during early adolescence are likely to produce a long-term impact on one's health outcomes (33). Healthy behaviors during childhood are the foundation of good oral health habits throughout life. A study showed that children under 5 years old had adequate cognitive ability to be managed dental caries by using a mobile application (16).

In this study, oral health education and behavioral intervention based on lifestyle were implemented. Previous studies on the ways of health education include traditional lecturing, experiential learning (26) and drama mode of health education by dental residents dressed mimicking cartoon characters (34). Oral health education can be led by dentists, schoolteachers, and social organizations. The systematic review showed that dentist-led health education was effective (35). Previous studies have shown that lifestyle-based behavioral intervention is effective for chronic disease management (36–38). The experimental group in this study achieved the integration of multiple self-management methods, including health education and lifestyle intervention, by relying on the online caries management platform. Chronic disease self-management has a positive and important impact on improving self-efficacy by setting goals for patients and helping them achieve goals. In this study, compared with traditional oral health management, the online caries management platform has advantages in improving children's oral health knowledge and behaviors, which to some extent reflects the improvement of patients' self-efficacy in caries management.

The index for caries in primary dentition (decayed-missing-filled surfaces index, dmfs) was not taken into consideration since many of the primary teeth were lost during the mixed dentition stage and it would bias the results (26). For this reason, only the permanent teeth that were present were considered for the calculation of the DMFS index. There was no significant difference in the caries status of the first permanent molars between the two groups before and after 1 year. This was similar to the results of a school-based oral health promotion programme conducted by Tai et al. in Wuhan, China, where mean DMFT was equal in two groups (24). Caries is a multifactorial chronic disease that requires a long period of observation. One year of follow-up may not be sufficient to observe its changes.

Compared with traditional lecturing, the online caries management platform can reduce the limitation of time and space on the communication between doctors and patients. According to the caries risk items of different individuals, the online caries management platform focuses on the implementation of oral health education in insufficient aspects. The oral health education strategy of the target population varies from person to person, and personalized health guidance can be achieved.

The effect of chronic disease management is mainly reflected in the change of various measuring indicators, which can be divided into clinical indicators and non-clinical indicators. Self-monitored physiological data and clinical examination results constitute clinical indicators. The behavior data of patients and the quality data of chronic disease management constitute non-clinical indicators. It is more and more convenient to use the Internet to obtain various measurement data of patients, and the accuracy and continuity are also gradually improved (39, 40). The online caries management platform provides a convenient and accurate way for oral health education and caries-related factor investigation (such as fluoride content of toothpaste, specific time of brushing, etc.), paving the way for caries-related lifestyle intervention.

The sample size and follow-up time of the study are limited, and a larger sample size and longer follow-up time are needed to evaluate the effect of this online platform on caries management. Since health literacy can be forgotten, the interval between implementing health education and evaluating participants' health literacy should be shortened in future studies. The participants in this study were all from the same city with poor population mobility. Random cluster sampling was conducted for them, and statistical analysis showed no difference in the education and socioeconomic status of their parents. The fluoride concentration of drinking water in the study area was not investigated and did not reach a suitable state. The study participants may intentionally provide more healthy attitude and behaviors compared with fact when filling out the questionnaire, which may lead to a bias. The physical and mental state of the study participants when filling out the questionnaire may affect the authenticity and accuracy of the items involved in the questionnaire, leading to a recall bias.

## 5. Conclusion

The online caries management platform has more advantages in improving oral health knowledge and behaviors than traditional lecturing. The online caries management platform provides a reliable way for the improvement of oral health-related behaviors. However, there were no significant differences in the caries status of the first permanent molars between the two groups at baseline and after a year. Caries is a chronic disease with multiple factors, which requires a longer follow-up.

## Data availability statement

The original contributions presented in the study are included in the article/Supplementary material, further inquiries can be directed to the corresponding author.

## Ethics statement

The studies involving human participants were reviewed and approved by Ethics Committee of Stomatological Hospital, Southern Medical University. Written informed consent to participate in this study was provided by the participants' legal guardian/next of kin. Written informed consent was obtained from the individual(s), and minor(s)' legal guardian/next of kin, for the publication of any potentially identifiable images or data included in this article.

## Author contributions

SY conceptualized the study, analyzed the findings, and wrote the manuscript. SY, SL, XY, LH, and XC searched the literature, designed studies, collected, analyzed data, and provided important scientific input. GQ supervised the whole study. All authors collaboratively discussed key decisions throughout the review, provided critical feedback on the preliminary manuscript, and gave final approval of the version to be published.
