# Supplementary material for: Effectiveness of online caries management platform in children's caries prevention: A randomized controlled trial
Source: Front Public Health. 2023 Feb 9;11:1102503. doi: 10.3389/fpubh.2023.1102503 (PMC9947237; doi:10.3389/fpubh.2023.1102503)
Supplement: Supplementary file 1 [file Table_1.DOC]

Supplementary Material

Table1. Sugar consumption behavior of the two groups at baseline (%)

| Factors | group | After a year | | χ^2^ | *P* |
| --- | --- | --- | --- | --- | --- |
|  |  | Experimental group | Control group |  |  |
| Does your child eat dessert or drink sweet drinks before bed? | every day | 21.93 | 27.03 | 4.652 | 0.098 |
|  | not every day | 60.53 | 64.86 |  | |
|  | seldom | 17.54 | 8.11 |  |  |
| Frequency of eating desserts and sweets | ≥ 2 times a day | 4.39 | 4.50 | 4.458^a^ | 0.486 |
|  | once a day | 6.14 | 13.51 |  | |
|  | once a week | 12.28 | 10.81 |  |  |
|  | 2 to 6 times a week | 31.58 | 34.23 |  |  |
|  | 1-3 times a month | 9.65 | 8.11 |  |  |
|  | rarely | 35.96 | 28.83 |  |  |
| Frequency of drinking sweet drinks (carbonated drinks, not freshly squeezed fruit juices) | ≥ 2 times a day | 2.63 | 0.90 | 1.961^a^ | 0.855 |
|  | once a day | 2.63 | 1.80 |  | |
|  | once a week | 15.79 | 17.12 |  |  |
|  | 2 to 6 times a week | 8.77 | 10.81 |  |  |
|  | 1-3 times a month | 14.91 | 18.02 |  |  |
|  | rarely | 55.26 | 51.35 |  |  |
| Frequency of drinking sweetened milk, yogurt, soy milk, milk powder, tea, coffee | ≥ 2 times a day | 3.51 | 1.80 | 4.371^a^ | 0.497 |
|  | once a day | 13.16 | 18.92 |  | |
|  | once a week | 8.77 | 10.81 |  |  |
|  | 2 to 6 times a week | 18.42 | 14.41 |  |  |
|  | 1-3 times a month | 11.40 | 6.31 |  |  |
|  | rarely | 44.74 | 47.74 |  |  |

a likelihood-ratio test

Table2. Oral hygiene behavior of the two groups at baseline (%)

| Factors | group | After a year | | χ^2^ | *P* |
| --- | --- | --- | --- | --- | --- |
|  |  | Experimental group | Control group |  |  |
| Frequency of using dental floss | every day | 0.88 | 0 | 3.129^a^ | 0.372 |
|  | every week | 0 | 0.90 |  | |
|  | occasionally use | 19.30 | 22.52 |  |  |
|  | don't know or never use it | 79.82 | 76.58 |  |  |
| Brushing times per day | ≥2 times per day | 56.14 | 60.36 | 1.143 | 0.565 |
|  | once a day | 39.47 | 33.33 |  | |
|  | <1 time a day | 4.39 | 6.31 |  |  |
| Time of brushing each time | >3min | 6.15 | 11.71 | 2.210 | 0.331 |
|  | 1-3min | 57.89 | 55.86 |  | |
|  | <1min | 35.96 | 32.43 |  |  |

a likelihood-ratio test
